# Histological Response and Prognostic Factors in Gastric Adenocarcinoma Treated With Fluorouracil, Leucovorin, Oxaliplatin, and Docetaxel (FLOT) Chemotherapy: A Retrospective Single-Center Study

**DOI:** 10.7759/cureus.100416

**Published:** 2025-12-30

**Authors:** Hugo Pereira, Daniel Martins, Ana Tavares, Andreia Amado, Amélia Tavares, Bela Pereira

**Affiliations:** 1 General Surgery, Unidade Local de Saúde Gaia e Espinho, Vila Nova de Gaia, PRT

**Keywords:** becker criteria, flot regimen, gastric cancer, neoadjuvant chemotherapy, surgical oncology, tumor regression

## Abstract

Background: Neoadjuvant chemotherapy is widely used in the management of resectable gastric adenocarcinoma and is considered an important component of multimodal treatment. This study provides real-world evidence on histological response patterns following fluorouracil, leucovorin, oxaliplatin, and docetaxel (FLOT) chemotherapy, suggesting that tumor biology may play a more relevant role than regimen selection in determining pathological response.

Methods: We conducted a retrospective study including 104 patients who underwent curative surgery for gastric adenocarcinoma between January 2015 and December 2024. Patients were divided into two groups according to the neoadjuvant chemotherapy regimen: FLOT or other protocols. Tumor regression was evaluated by the Becker criteria. Clinicopathological variables were analyzed to identify predictors of histological response.

Results: Of the 104 patients, 68 received the FLOT regimen, while 36 received alternative regimens. There was no statistically significant difference in the Becker regression between the FLOT and non-FLOT groups (p = 0.08). Within the FLOT cohort, factors associated with poorer tumor regression included poor differentiation, advanced T stage, nodal metastasis, lymphatic and perineural invasion (all p<0.05). Poorer histological response correlated with higher recurrence rates and shorter disease-free survival.

Conclusions: The type of neoadjuvant chemotherapy did not significantly influence Becker regression in this cohort. Tumor biology and pathological staging remain key determinants of response. These findings reinforce the importance of individualized treatment strategies and suggest a role for biomarkers to guide therapeutic decisions in gastric cancer.

## Introduction

Gastric adenocarcinoma remains a major global health burden, ranking as the fifth most commonly diagnosed cancer and the third leading cause of cancer-related mortality [1]. In 2020 alone, nearly one million new cases and over 769,000 deaths were reported, underscoring the aggressive nature and poor prognosis of this malignancy [1]. The incidence of gastric adenocarcinoma shows significant geographical variation, with the highest rates observed in East Asia, particularly Japan and South Korea [2]. Several risk factors contribute to the development of gastric cancer, including dietary habits, chronic *Helicobacter pylori* infection, and genetic predisposition [3-5].

In recent years, the treatment of resectable gastric cancer has evolved significantly, with neoadjuvant chemotherapy emerging as a key component of multimodal strategies. This approach aims to downstage tumors, enhance the likelihood of achieving R0 resection, and address micrometastatic disease at an early stage [6]. Among the available regimens, the fluorouracil, leucovorin, oxaliplatin, and docetaxel (FLOT) regimen has become the standard of care, following the results of the FLOT-4 trial, which demonstrated superior overall and progression-free survival compared to epirubicin, cisplatin, and capecitabine/fluorouracil (ECF/ECX) regimens [7]. These benefits are attributed to the synergistic effects of the agents involved, enhancing tumor response and reducing recurrence.

Despite its widespread adoption, the impact of FLOT on histological tumor regression remains less well defined. Tumor regression, typically assessed using the Becker criteria, has emerged as a potential prognostic marker, with better regression correlating with improved oncological outcomes [8]. Becker regression evaluates the proportion of residual tumor within a fibrotic stroma following neoadjuvant therapy, providing histological insight into treatment response.

This study aimed to assess whether FLOT chemotherapy results in better histological regression, as assessed by the Becker criteria, compared to other regimens, and to identify clinicopathological factors influencing treatment response.

## Materials and methods

A retrospective study was conducted involving patients who underwent curative (R0) radical resection for gastric adenocarcinoma at Unidade Local de Saúde Gaia e Espinho, Portugal, between January 2015 and December 2024 following neoadjuvant chemotherapy. Patients were divided into two groups according to the treatment protocol: those receiving the FLOT regimen and those treated with alternative chemotherapy regimens.

Pathological tumor regression was assessed using the Becker criteria [8] based on histological examination of the resected specimens. Responses were classified as “good” (grades Ia-II) or “poor” (grade III) according to the percentage of residual tumor cells.

Clinicopathological variables analyzed included age, sex, preoperative performance status [9], preoperative laboratory values, clinical and pathological tumor-node-metastasis (TNM) classification (cTNM and pTNM) according to the American Joint Committee on Cancer (AJCC) Eighth Edition [10], histological differentiation, lymphovascular and perineural invasion, and treatment details.

Associations between categorical variables were analyzed using the chi-square test [11], and continuous variables were compared using the Mann-Whitney U test [12]. Overall survival (OS) and recurrence-free survival (RFS) were estimated using the Kaplan-Meier method [13], with group differences assessed by the log-rank test [14]. Median follow-up time was calculated using the reverse Kaplan-Meier method. Hazard ratios (HRs) and 95% confidence intervals (CIs) were obtained from Cox proportional hazards models.

Statistical analyses were performed using IBM SPSS Statistics software, version 24 (IBM Corp., Armonk, NY, USA), with statistical significance set at p<0.05. In accordance with institutional policy, this retrospective study, based exclusively on anonymized data, did not require formal written ethics committee approval or informed consent.

The standard FLOT regimen consisted of four preoperative and four postoperative cycles of fluorouracil (2600 mg/m²), leucovorin (200 mg/m²), oxaliplatin (85 mg/m²), and docetaxel (50 mg/m²) administered every two weeks. Alternative regimens included ECF/ECX, S-1 and oxaliplatin (SOX), or capecitabine and oxaliplatin (CAPOX), depending on the treating oncologist’s decision and patient tolerance. The median number of preoperative cycles completed was eight (range six to eight) in the FLOT group and six (range four to eight) in the non-FLOT group. No patients received neoadjuvant radiotherapy. Postoperative adjuvant therapy followed institutional protocols and was based on performance status, pathological stage, and tolerance to preoperative chemotherapy.

The primary endpoint of this study was histological tumor regression according to the Becker criteria. Secondary endpoints included RFS and OS.

## Results

A total of 104 patients were included in this study. Baseline characteristics are summarized in Table 1. Patients receiving FLOT were significantly younger than those treated with other neoadjuvant regimens (median 61.5 vs. 67.0 years, p = 0.012). No significant differences were observed between groups regarding sex distribution, comorbidities, BMI, hemoglobin, or albumin levels. Clinical staging parameters (cT and cN) also did not differ significantly between the treatment groups.

**Table 1:** Baseline characteristics of the study population Data are presented as medians or frequencies (%). Statistical comparisons were performed using the Mann-Whitney U test [12] for continuous variables and chi-square tests [11] for categorical variables. Clinical staging parameters (cT and cN) were classified according to the American Joint Committee on Cancer (AJCC) Eighth Edition [10]. "Stage distribution” indicates that cT and cN were analyzed as multi-category variables, and the reported p-values reflect comparisons of their full stage distributions rather than a single summary measure.

Variable	FLOT (n = 68)	Other regimen (n = 36)	Test statistic	p-value
Age (years), median	61.5	67	Mann-Whitney U = 856.5	0.012
Sex male, n (%)	42 (61.8%)	28 (77.8%)	χ²(1) = 2.06	0.151
Hypertension, n (%)	30 (44.1%)	20 (55.6%)	χ²(1) = 0.70	0.403
Diabetes, n (%)	18 (26.5%)	11 (30.6%)	χ²(1) = 0.07	0.79
Pulmonary disease, n (%)	3 (4.4%)	1 (2.8%)	χ²(1) = 0.15	0.701
BMI (kg/m²), median	24.29	24.63	Mann-Whitney U = 1294.5	0.632
Hemoglobin (g/dL), median	12.4	11.95	Mann-Whitney U = 1188.5	0.906
Albumin (g/dL), median	4.2	4.1	Mann-Whitney U = 1496.5	0.062
Clinical T stage (cT)	Stage distribution	Stage distribution	χ²(3) = 6.68	0.083
Clinical N stage (cN)	Stage distribution	Stage distribution	χ²(1) = 2.65	0.104

The median follow-up time was 34.4 months (interquartile range 21.9-62.6 months). The majority were male (67.3%), with a mean age of 62 years. Among the 68 patients who received FLOT chemotherapy, baseline characteristics are detailed in Table 2. The remaining 36 patients (35%) were treated with other chemotherapy regimens.

**Table 2:** Clinicopathological features in the FLOT group Characteristics of the variables analyzed (FLOT group, n=68). Tumor staging parameters (cT, pT, pN) were classified according to the American Joint Committee on Cancer (AJCC) Eighth Edition [10]. Histological tumor regression was graded using the Becker classification [8]. FLOT: fluorouracil, leucovorin, oxaliplatin, and docetaxel; cT: clinical T stage; pT: pathological T stage; pN: lymph node metastasis

Variable	n (%)
Mean age (years)	60
Male gender	70 (67.3%)
cT1-T2	18 (26.5%)
cT3-T4	50 (73.5%)
N0	23 (33.8%)
Elevated preoperative CEA	25 (36.8%)
Elevated preoperative CA 19.9	26 (38.2%)
pT0-T2	29 (42.6%)
pT3-T4	39 (57.4%)
pN0	31 (45.6%)
Lymphatic invasion	50 (58.8%)
Perineural invasion	33 (48.5%)
Vascular invasion	25 (35.3%)
Becker grades I-II	43 (63.2%)
Becker grade III	25 (36.8%)
Recurrence	24 (35.3%)
Death	20 (29.4%)
Cancer-related death	18 (26.5%)

When comparing the two groups, no statistically significant difference in the Becker regression was observed between patients treated with FLOT and those receiving alternative regimens (p = 0.08). In the FLOT group, 63.2% of patients achieved Becker grades I-II, while 36.8% had grade III regression. In the non-FLOT group, 50% achieved grades I-II and 50% grade III.

Focusing exclusively on the FLOT subgroup, several clinicopathological variables showed significant associations with tumor regression. Well-differentiated tumors demonstrated a more favorable response to neoadjuvant treatment, with a significant association between tumor differentiation and Becker regression (p = 0.009). Clinical T stage (cT) also correlated with treatment response, with more advanced tumors (T3-T4) exhibiting poorer regression (p = 0.04). Pathological T stage (pT) showed a similar pattern, with more advanced tumors demonstrating less histological regression (p = 0.003). The absence of lymph node metastasis (pN0) correlated with a better response compared to patients with positive lymph nodes (pN1) (p = 0.014). These findings are summarized in Table 3.

**Table 3:** Comparison of variables between good and poor histological responders All p-values from chi-square tests [11] comparing good vs. poor histological response (Becker I-II vs. III) [8] within the FLOT group. Tumor staging parameters (cT, pT, pN) were classified according to the American Joint Committee on Cancer (AJCC) Eighth Edition [10]. FLOT: fluorouracil, leucovorin, oxaliplatin, and docetaxel

Variable	Test statistic (df), p-value
Tumor differentiation	χ²(1) = 3.20 p = 0.01
Initial clinical T stage (cT)	χ²(3) = 14.89, p = 0.04
Pathological T stage (pT)	χ²(4) = 16.14, p = 0.00
Lymph node metastasis (pN)	χ²(3) = 10.60, p = 0.01
Lymphatic invasion	χ²(1) = 12.05, p = 0.00
Perineural invasion	χ²(1) = 17.73, p = 0.00
CA 19.9 (normal vs. high)	χ²(1) = 0.24, p = 0.626

Additionally, both lymphatic invasion (p = 0.001) and perineural invasion (p < 0.0001) were strongly associated with poorer tumor regression. Elevated preoperative CA 19.9 levels were more frequently observed in patients with poorer response, although this did not reach statistical significance (p = 0.626).

Regarding oncological outcomes, patients with poorer histological response (Becker grade III) showed shorter median disease-free survival compared to good responders, although this difference did not reach statistical significance. Median time to recurrence was 15 months in good responders and nine months in poor responders. In contrast, OS differed significantly between groups, with patients demonstrating good histological response achieving a median survival of 16.6 months compared to 14.3 months in poor responders. These results are summarized in Table 4.

**Table 4:** Survival outcomes according to histological response Survival outcomes according to histological response (FLOT group). Histological tumor regression was classified using the Becker grading system [8]. FLOT: fluorouracil, leucovorin, oxaliplatin, and docetaxel

Outcome	Grouping	Test	Statistic	p-value
Overall survival	Good vs. poor Becker response	Log-rank	χ²(1) = 5.32	0.021
Recurrence-free survival	Good vs. poor Becker response	Log-rank	χ²(1) = 0.44	0.506

To further illustrate these findings, Kaplan-Meier survival curves were generated to compare RFS and OS between patients with good (Becker I-II) and poor (Becker III) histological response within the FLOT group.

**Figure 1:**
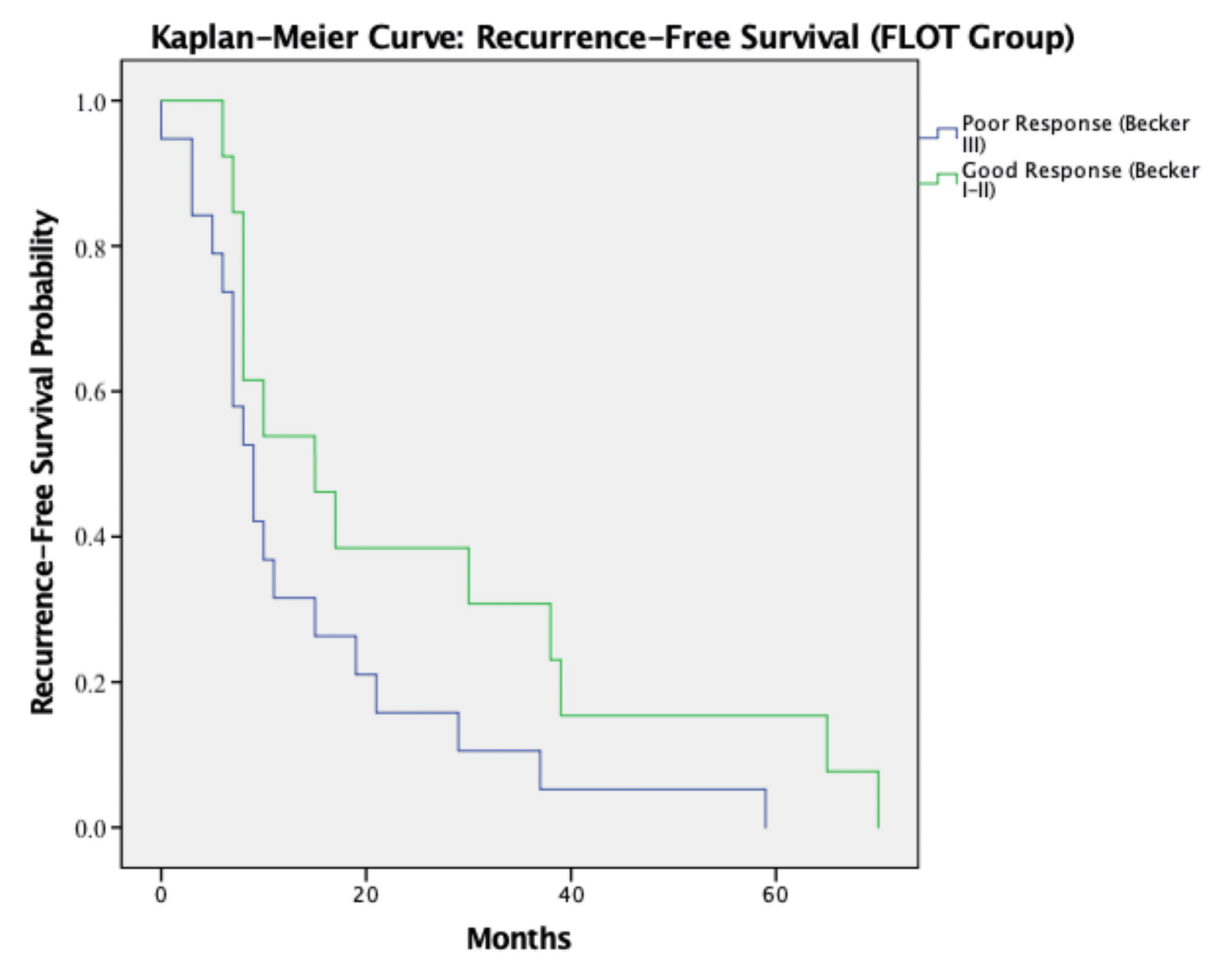
Recurrence-free survival curve Kaplan-Meier curve [13] for recurrence-free survival stratified by Becker response (FLOT group) [8]. Log-rank test: χ²(1) = 0.44, p = 0.506 [14]. FLOT: fluorouracil, leucovorin, oxaliplatin, and docetaxel

Patients with good histological response (Becker I-II) demonstrated a median RFS of 15 months, compared to nine months in poor responders (Becker III), representing a difference of six months in favor of those with better pathological response. Although numerically longer, this difference did not reach statistical significance (log-rank test, p = 0.506) (Figure 1).

**Figure 2:**
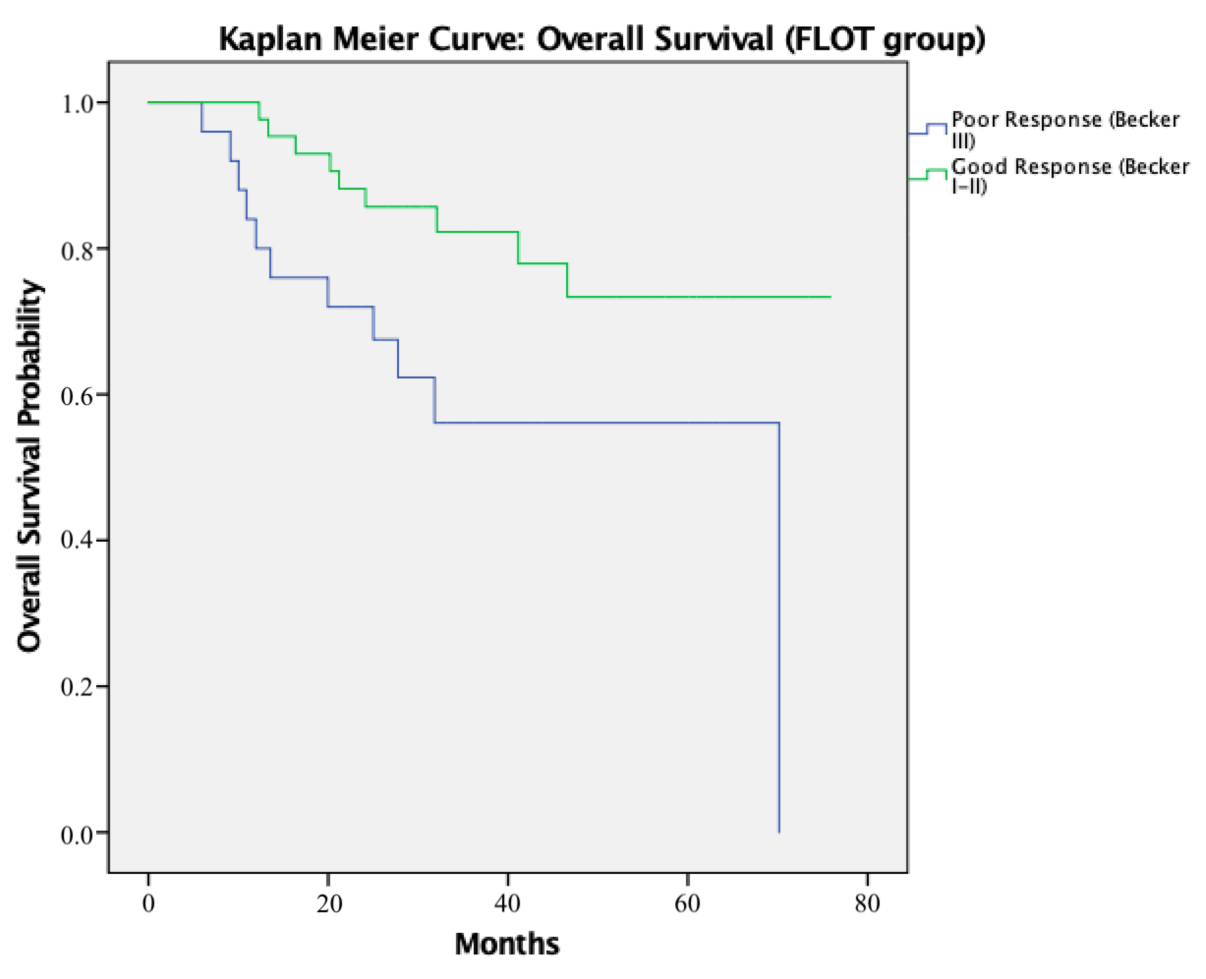
Overall survival curve Kaplan-Meier curve [13] for overall survival stratified by Becker response (FLOT group) [8]. Log-rank test: χ²(1) = 5.32, p = 0.021 [14]. FLOT: fluorouracil, leucovorin, oxaliplatin, and docetaxel

Similarly, Kaplan-Meier survival curves were generated to assess OS according to histological response. Patients with good histological response (Becker I-II) demonstrated a median OS of 16.6 months (95% CI: 4.4-25.5), compared to 14.3 months (95% CI: 6.1-11.8) in poor responders (Becker III), reflecting a difference of 2.3 months. This difference was statistically significant (log-rank test, p = 0.021) (Figure 2).

A separate comparison of survival outcomes between treatment groups was performed for completeness; however, interpretation was limited by heterogeneity in baseline characteristics.

In this cohort, tumor differentiation, staging, lymph node involvement, and the presence of lymphatic and perineural invasion emerged as significant prognostic factors influencing histological response in patients treated with the FLOT regimen. Although patients with poor pathological response tended to experience earlier recurrence, this difference was not statistically significant. In contrast, poor responders demonstrated notably shorter OS compared to those achieving good histological regression. The relatively modest survival outcomes observed in this study likely reflect real-world factors, including more advanced disease at presentation, variability in postoperative treatment completion, and the inherent limitations of a retrospective single-center analysis, such as variable follow-up duration and differing adherence to treatment pathways.

## Discussion

The results of this study demonstrate that, despite the recognized efficacy and widespread adoption of the FLOT chemotherapy regimen for gastric adenocarcinoma, no statistically significant difference in histological tumor regression according to Becker grading was observed when compared to other chemotherapy regimens. A non-significant trend toward improved Becker regression was observed in the FLOT group (p = 0.08). Given the relatively small sample size (n = 104; 68 in the FLOT group and 36 in the non-FLOT group), this study was likely underpowered to detect modest differences in pathological response between treatment strategies. Nevertheless, it provides real-world insight into treatment response patterns and prognostic factors in gastric adenocarcinoma.

Although previous trials have demonstrated survival benefits with FLOT, our study was not designed or powered to compare OS between treatment regimens, and such analyses were therefore exploratory [15]. The absence of a significant difference in histological regression between regimens likely reflects patient heterogeneity, underlying differences in tumor biology, and inherent variability in chemotherapy response observed in routine clinical practice [16].

It is important to acknowledge that patients receiving FLOT chemotherapy were statistically younger than those treated with alternative regimens. Although this difference reached statistical significance, the absolute difference in median age was modest and occurred within a similar clinical age range typical for gastric adenocarcinoma. Moreover, no significant differences were observed between groups regarding comorbidity burden, nutritional parameters, or clinical staging. Nevertheless, age may have influenced treatment selection in routine clinical practice, and this imbalance represents a source of selection bias that limits the interpretation of direct comparisons between chemotherapy regimens. Consequently, comparative analyses should be regarded as exploratory.

Within the FLOT subgroup, several prognostic factors were significantly associated with tumor regression. Although well-differentiated tumors showed a trend toward better histological response, this association did not reach statistical significance in our cohort. Previous studies have suggested that tumor differentiation may influence chemotherapy sensitivity [17], whereas poorly differentiated tumors, typically more aggressive, have been associated with resistance to treatment [18]. Tumor staging also played a crucial prognostic role; patients with advanced T stage exhibited less histological regression, reinforcing the importance of accurate preoperative staging to guide therapeutic strategies [19,20]. Furthermore, the absence of lymph node metastases (pN0) correlated with better tumor regression, supporting the established prognostic value of nodal status in gastric cancer [21,22]. These findings should be interpreted cautiously, as multivariate adjustment was not performed and several variables are biologically and statistically interrelated.

Lymphatic and perineural invasion were also strongly associated with poorer histological response. These pathological features reflect both local tumor aggressiveness and potential for systemic dissemination, factors known to reduce chemotherapy efficacy [23]. Collectively, these findings underscore the influence of tumor biology on treatment response, beyond the choice of chemotherapy regimen.

An important clinical consideration arising from these findings concerns the current practice of maintaining postoperative FLOT chemotherapy in patients who exhibit poor histological response after neoadjuvant treatment. Although the FLOT-4 trial established perioperative FLOT as a standard of care [7], our data suggest that pathological response - or lack thereof - provides critical prognostic information. These findings raise the question of whether postoperative treatment should be individualized according to pathological response.

In patients with poor response, early adaptation of treatment strategies, including the integration of novel therapies such as immunotherapy or targeted agents, might be more beneficial. Prospective studies are needed to clarify whether maintaining FLOT postoperatively in poor responders offers any additional survival advantage or whether treatment should be tailored based on tumor biology and histological response.

Potential Explanations for These Findings

Several factors may contribute to the absence of a significant difference in the Becker regression between FLOT and non-FLOT regimens in our study.

Firstly, the heterogeneity of chemotherapy protocols in the non-FLOT group may have confounded the results, potentially masking more subtle differences between treatment strategies. Future studies should aim to compare FLOT with more homogeneous treatment regimens to better isolate its impact on tumor regression [24].

Secondly, other prognostic factors not assessed in this study may influence tumor response. Emerging evidence suggests that molecular biomarkers such as HER2 and PD-L1 expression, immune response characteristics, and patients' nutritional status can impact the efficacy of chemotherapy and histological regression [25]. Further research is warranted to explore these factors in the context of neoadjuvant treatment response.

Finally, the duration of neoadjuvant chemotherapy may also play a role in tumor regression. Although the FLOT regimen is standardized in clinical trials, real-world treatment durations can vary due to patient tolerance or clinical decisions. Studies evaluating the optimal duration of neoadjuvant therapy could provide valuable insights into whether extending or shortening treatment influences histological response and long-term outcomes [26].

Limitations

This study is subject to several limitations inherent to retrospective analyses, including potential selection bias, heterogeneity within the patient population, and variability in chemotherapy regimens used in the control group. The relatively small sample size (n = 104; 68 in the FLOT group and 36 in the non-FLOT group) likely reduced the ability to detect modest differences in histological regression or survival outcomes. Additionally, the single-center design introduces variability in follow-up duration and treatment adherence, which may influence both pathological and clinical endpoints.

Becker regression assessment is subjective and operator-dependent, adding another source of variability in evaluating tumor response. Multivariate analysis was not performed due to the limited sample size and potential collinearity among pathological variables, meaning that the reported associations rely on univariable analyses and should be interpreted with caution. Despite these constraints, the study provides real-world insight into treatment response patterns and highlights the need for refining therapeutic strategies, including consideration of postoperative adaptations in patients with limited pathological regression. Future work incorporating molecular and genetic profiling, as well as emerging therapeutic modalities, may help identify individuals less likely to benefit from standard regimens and support more personalized treatment pathways [27].

Future Directions

Future prospective studies should aim to overcome the limitations of retrospective analyses by employing standardized evaluation criteria and enrolling larger, more homogeneous patient cohorts. Incorporating molecular and genetic profiling into treatment strategies may enhance the identification of patients more likely to benefit from FLOT chemotherapy or alternative therapies. Additionally, the exploration of combination therapies, particularly those involving immunotherapy, holds promise for improving treatment outcomes in patients whose tumors exhibit poor regression following standard chemotherapy [28].

## Conclusions

In this study, no significant difference was observed in histological tumor regression, assessed by Becker grading, between patients with gastric adenocarcinoma treated with the FLOT chemotherapy regimen and those receiving alternative neoadjuvant therapies. The findings suggest that tumor response is influenced by multiple clinicopathological factors, including differentiation, staging, lymph node involvement, and lymphovascular or perineural invasion. These characteristics emerged as important prognostic indicators and reinforce the importance of tailoring treatment strategies to individual tumor biology rather than relying solely on the selected chemotherapy protocol. Given the limited sample size, the ability to detect modest differences in regression or survival was restricted, but the analysis still offers meaningful real-world insight into response patterns and prognostic determinants.

Although the overall outcomes in this cohort varied, the data indicate that not all patients experience substantial histological regression with commonly used regimens, highlighting the need to refine treatment approaches for subgroups with poor pathological response. Variability related to patient selection, disease stage, treatment adherence, and follow-up duration also underscores the challenges inherent to retrospective analyses. Despite these limitations, the study emphasizes the value of integrating pathological features into treatment planning and supports further investigation into postoperative strategies, molecular profiling, and newer therapeutic options. Future prospective research with larger and more homogeneous cohorts is essential to validate these observations and improve the precision of neoadjuvant treatment selection in gastric adenocarcinoma.
